# Projections from the Rostral Zona Incerta to the Thalamic Paraventricular Nucleus Mediate Nociceptive Neurotransmission in Mice

**DOI:** 10.3390/metabo13020226

**Published:** 2023-02-03

**Authors:** Feng-Ling Wu, Si-Hai Chen, Jia-Ni Li, Liu-Jie Zhao, Xue-Mei Wu, Jie Hong, Ke-Hua Zhu, Han-Xue Sun, Su-Juan Shi, E Mao, Wei-Dong Zang, Jing Cao, Zhen-Zhen Kou, Yun-Qing Li

**Affiliations:** 1 Department of Human Anatomy, College of Preclinical Medical Sciences, Zhengzhou University, Zhengzhou 450001, China; 2 Department of Anatomy, Histology and Embryology and K. K. Leung Brain Research Centre, The Fourth Military Medical University, Xi’an 710032, China; 3 Department of Human Anatomy, The School of Basic Medical Sciences, Fujian Medical University, Fuzhou 350122, China; 4 Department of Human Anatomy, Baotou Medical College Inner Mongolia University of Science and Technology, Baotou 014040, China; 5 Institute of Medical Research, Northwestern Polytechnical University, Xi’an 710072, China; 6 Department of Geriatrics, Tangdu Hospital, The Fourth Military Medical University, Xi’an 710038, China; 7 Key Laboratory of Brain Science Research and Transformation in Tropical Environment of Hainan Province, Hainan Medical University, Haikou 571199, China; 8 Department of Anatomy, College of Basic Medicine, Dali University, Dali 671000, China

**Keywords:** zona incerta, gamma–aminobutyric acid-ergic, thalamic paraventricular nucleus, GABA-A receptor, nociceptive neurotransmission

## Abstract

Zona incerta (ZI) is an integrative subthalamic region in nociceptive neurotransmission. Previous studies demonstrated that the rostral ZI (ZIR) is an important gamma–aminobutyric acid-ergic (GABAergic) source to the thalamic paraventricular nucleus (PVT), but whether the ZIR–PVT pathway participates in nociceptive modulation is still unclear. Therefore, our investigation utilized anatomical tracing, fiber photometry, chemogenetic, optogenetic and local pharmacological approaches to investigate the roles of the ZIR^GABA+^–PVT pathway in nociceptive neurotransmission in mice. We found that projections from the GABAergic neurons in ZIR to PVT were involved in nociceptive neurotransmission. Furthermore, chemogenetic and optogenetic activation of the ZIR^GABA+^–PVT pathway alleviates pain, whereas inhibiting the activities of the ZIR^GABA+^-PVT circuit induces mechanical hypersensitivity and partial heat hyperalgesia. Importantly, in vivo pharmacology combined with optogenetics revealed that the GABA-A receptor (GABA_A_R) is crucial for GABAergic inhibition from ZIR to PVT. Our data suggest that the ZIR^GABA+^–PVT pathway acts through GABA_A_R-expressing glutamatergic neurons in PVT mediates nociceptive neurotransmission.

## 1. Introduction

Pain is a complex sensory experience that has been well recognized as a major clinical, social, and economic problem. Although considerable progress has been made in elucidating the mechanisms of pain, studies about the basic pathway and circuit of the nervous system are still in progress [1,2].

As the hypothalamic region of the thalamus, zona incerta (ZI) contains mostly inhibitory neurons, including gamma–aminobutyric acid-ergic (GABAergic) neurons [3]. ZI is roughly divided into four components: the rostral ZI (ZIR), the dorsal ZI (ZID), the ventral ZI (ZIV) and the caudal ZI (ZIC). Distinct ZI components display diverse connectivity patterns and play multiple roles [4]. Studies have shown that the ZI is involved in a variety of functions such as defensive behaviors [5], feeding and drinking [6], sleep and circadian rhythms [7]. 

Morphological evidence reveals that the spinothalamic tract sends dense nociceptive inputs to ZI, suggesting a potential role of ZI in nociceptive neurotransmission [8,9]. In addition, the activities of GABAergic neurons within ZI were decreased in chronic pain rodents compared with the sham-treated group [10,11,12]. On the other hand, the ZI deep brain stimulation with 20 Hz decreased pain in humans [13]. These reports indicate that as a key nucleus, ZI might be involved in the pain neurotransmission and modulation. Previous literature has paid attention to the ZIV involvement in the regulation of pain information [14]. However, except for ZIV, different parts of ZI may respond differently to nociceptive stimuli, the roles of other parts of ZI in nociceptive neurotransmission are still needed to be elucidated.

The thalamic paraventricular nucleus (PVT), one of important thalamic midline nucleus, is mainly composed of glutamatergic neurons [15]. Previous research found that inhibition of PVT activities could alleviate visceral and neuropathic pain [16,17]. Since the PVT is lacks of the GABAergic inhibitory neurons, the modulation of PVT function relies on inhibitory afferents for excitation and inhibition balance in neurotransmission [18]. It is noted that ZIR is one of GABAergic source to PVT [19], recent studies demonstrated that the GABAergic ZIR neurons project to PVT neurons and contribute to conditioned feeding behavior [6,20]. However, the roles of the ZIR–PVT pathway in nociceptive neurotransmission have not been fully addressed. 

Based on these considerations, we hypothesized that the GABAergic ZIR neurons project to PVT and participate in pain. In the present study, anatomical, chemogenetic, optogenetic and pharmacological approaches were applied to investigate the roles of the ZIR^GABA+^ –PVT pathway in nociceptive neurotransmission. 

## 2. Materials and Methods

### 2.1. Animals

Experiments were performed with adult male (6–12-week-old) wild-type (WT) C57BL/6J (purchased from the Laboratory Animal Center of the Fourth Military Medical University), GAD2-Cre (JAX#010802), Ai9-TdTomato (JAX#007909) mice (purchased from Jackson Laboratories) and GAD67-GFP. About 200 mice were used in the study, and the number of the mice per subgroups is described in the figure legends. All animals used were housed in a 12 h light/dark cycle at 22–25 °C with food and water given ad libitum. Animal care and used strictly followed institutional guidelines and governmental regulations. All protocols to reduce the suffer of the animal from surgical operation were performed in accordance with the Animal Care and Use Committees at The Fourth Military Medical University (NO: IACUC-20210356).

### 2.2. Stereotaxic Injections 

Mice were anesthetized with intraperitoneal (*i.p.*) injection of pentobarbital sodium (40 mg/kg). Mice were then fixed on a stereotaxic apparatus (RWD Life Science, Shenzhen, China), and were stereotactically injected with fluorescent tracers or viruses through a glass micropipette (internal tip diameter 15–25 µm) attached to a 1 µL Hamilton microsyringe. Following injection, the needle was left in place for another 10 min before retraction.

The injection coordinates are listed as follows based on brain atlas: ZIR: anterior posterior (AP), −1; medial lateral (ML), 0.7; and dorsal ventral (DV), −4.67 mm. PVT: AP, −0.94; ML, 0; and DV, −3.25 mm. All tracers and viruses were injected by pressure through a microsyringe with a constant flow pump at a speed of 20 nl/min. All viruses were purchased from by BrainVTA Co., Ltd (Wuhan, China).

The rAAV2/9-hSyn-eGFP-WPRE-hGH pA (100 nl, 2 × 10^12^ vg/mL, PT-1990) was injected into the right ZIR in GAD2-Ai9 mice and the rAAV2/9-EF1a-DIO-mcherry-WPRE-hGH pA (100 nl, 1.1 × 10^13^ vg/mL, PT-0013) was injected into the right ZIR in GAD2-Cre mice for tracing studies.

For fiber photometry, the rAAV2/1-hSyn-Cre-WPRE-hGH pA (100 nl, 1.18 × 10^13^ vg/mL, PT-0136) was injected into the bilateral ZIR and the rAAV-CAG-FLEX-jGCaMP7s-WPRE-SV40 pA (200 nl, 2 × 10^12^ vg/mL, PT-1421) was injected into the PVT of C57BL/6J mice to label ZI-projecting PVT neurons. Three weeks after the virus were injected, the fibers (200 µm OD, 0.37 NA, Fiblaser, Shanghai, China) were implanted above the PVT 0.1 mm. 

For chemogenetic manipulation, the rAAV2/9-EF1a-DIO-hM3D(Gq)-EYFP-WPRE-hGH pA (100 nl, 5.2 × 10^12^ vg/mL, PT-0816), rAAV2/9-EF1a-DIO-hM4D(Gi)-EYFP-WPREs (100 nl, 5.04 × 10^12^ vg/mL, PT-0815) or the control rAAV2/9-EF1a-DIO-EYFP-WPRE-hGH pA (100 nl, 5 × 10^12^ vg/mL, PT-0012) were injected into the bilateral ZIR of GAD2-Cre mice to specifically manipulate ZIR neurons. 

For optogenetic manipulate the ZIR^GABA+^–PVT pathway, the rAAV2/9-EF1a-DIO-hChR2(H134R)-EYFP-WPRE pA (100 nl, 2.64 × 10^12^ vg/mL, PT-0001), rAAV2/9-EF1a-DIO-eNpHR3.0-EYFP-WPRE pA (100 nl, 2.21 × 10^12^ vg/mL, PT-0006) or the control rAAV2/9-EF1a-DIO-EYFP-WPRE pA (100 nl, 5 × 10^12^ vg/mL, PT-0012) were injected into the bilateral ZIR of GAD2-Cre mice. Three weeks later, the opto fibers were implanted above PVT.

The rAAV2/R -hSyn-Cre-WPRE-hGH pA (200 nl, 2.93 × 10^12^ vg/mL, PT-0136) was injected into the PVT and the rAAV2/9-VGAT1-DIO-hM3D(Gq)-mcherry-WPREs (100 nl, 3.6 × 10^12^ vg/mL, PT-0153) was injected into the bilateral ZIR to specifically activate the ZIR^GABA+^–PVT pathway. The rAAV2/9-VGAT1-DIO-mcherry-WPRE pA (100 nl, 2 × 10^12^ vg/mL, PT-3325) was used as control.

For microinjection experiments, mice were implanted with a cannula (RWD Life Science, China) above the PVT for infusion of GABA_A_R antagonists bicuculline (0.1 μg). The microinjector was 0.5 mm longer than the guide sleeve and was attached to a polyethylene tube that was attached to a 10 μL syringe. The drug was infused into the PVT over 2–3 min. The microinjector was left for a further 1 min to allow the drug to diffuse. The mice were placed in the experimental apparatus 10 min after administration connect optical fibers.

For the neural tracing, the tracers used in this study include the retrograde tracers CTB (200 nl, Sigma, Louis, MO, USA) and 594 retroBeads (200 nl, Lumafluor, New York, NY, USA) that were stereotaxically injected into PVT. After surgery, mice were allowed to survive for 7–10 day.

### 2.3. Histology

Animals were deeply anesthetized with excess pentobarbital sodium (100 mg/kg, *i.p.*) and transcardially perfused with 50 mL of 0.01 M phosphate-buffered saline (PBS, pH 7.4), followed by 150 mL of 4% paraformaldehyde in 0.1 M phosphate buffer (PB, pH 7.4) to fix the tissue. Brains were removed and soaked in 4% PFA for 2–4 h. Subsequently immersed into 30% sucrose dissolved in 0.1 M PB until they sunk to the bottom of the containers. The brains were cut into 30 µm transverse sections (CM1950; Leica, Heidelberg, Germany) and stored in PBS at 4 °C until immunostaining processed. All sections were blocked in 5% donkey serum for 30 min and then incubated with the primary antibodies at room temperature for 16–24 h and the secondary antibodies at room temperature for 4–6 h. 

For CTB-labeled neurons, the primary antibody was goat anti-CTB (1:500, Listlabs, Santa Clara, CA, USA) and the secondary antibodies were donkey Alexa 594 donkey anti-goat (1:500, A31571, Invitrogen, Carlsbad, CA, USA).

For FOS neurons induced by formalin, the mice were injected with 4% formalin (20 μL) in the hind paw and then were placed into original rearing cages for 2 h before being perfused. The primary antibody was mouse anti-FOS (1:500, ab11959, Abcam, Cambridge, MA, USA). The secondary antibodies were donkey Alexa 647 donkey anti-mouse (1:500, A31571, Invitrogen).

Immunostaining methods were used to evaluate the triple-labeling of mcherry/CaMKII/GABA_A_R in PVT. The primary antibodies were guinea pig anti-GABA_A_R (1:200, A Gp-083, Alomone, Jerusalem, Israel) and rabbit anti-CaMKII (1:500, ab134041, Abcam). The secondary antibodies were donkey Alexa 488 donkey anti-guinea pig and Alexa 647 donkey anti-rabbit (1:500, A31571, Invitrogen).

### 2.4. Whole-Cell Patch-Clamp Recordings

Mice were anesthetized with pentobarbital sodium (40 mg/kg, *i.p.*). Coronal slices (300 µm thick) containing the ZIR were cut on a vibrating microtome (Leica VT 1200s, Heidelberger, Nussloch, Germany) at 4°C. Slices were then transferred to a submerged recovery chamber containing oxygenated artificial cerebrospinal fluid (ACSF) consisting of (in mM) 124 NaCl, 25 NaHCO_3_, 2.5 KCl, 1 NaH_2_PO_4_, 2 CaCl_2_, 1 MgSO_4_ and 10 glucose saturated with 95% O_2_ and 5% CO_2_. The recordings were performed in voltage-clamp or current-clamp mode using an Axon 700B amplifier (Molecular Devices). To test the effectiveness of the ChR2 viruses, optical stimulation (473 nm) was applied with a custom laser fiber. The stimulation pattern was similar as that used in vivo (5 Hz, 10 Hz, and 20 Hz pulse trains). To test the effectiveness of the ZIR^GABA+^-PVT virus projection, clozapine evoked currents were recorded in voltage-clamp mode with membrane potential held at 70 mV, and cells were stimulated using 20 μM clozapine.

### 2.5. Fiber Photometry

A fiber photometry system (Inper, Hangzhou, China) was used to record the fluorescence signals generated by 470 nm LED light and 410 nm LED light excitation. On the day of the experiment, mice were acclimated for 30 min in the behavioral test room, and basal fluorescence was recorded for 5 min after acclimation. Next, mice were stimulated with *von* Frey filament (0.07 g, 1.0 g) and shocked (0.5 s, 0.3 mA). For data analysis, fluorescence change (delta*F/F*) was represented by (*F* − *F*0)/*F*0 and the *F0* is the averaged fluorescence in baseline period. Mice without the correct viral transduction or the correct fiber-optic site were excluded from the analysis.

### 2.6. Optogenetic Manipulation

For activating the ZIR^GABA+^–PVT pathway, a 473 nm laser (20 Hz, 5–10 mW, 10 ms pulse duration) was delivered. For inhibition of the ZIR^GABA+^–PVT pathway, a constant laser (589 nm, 5 mW) was delivered.

### 2.7. Chemogenetic Manipulation

All behavioral tests were performed 30 min after intraperitoneal injection of 0.1 mg/kg clozapine (Sigma) in chemogenetic manipulation. The different behavior experiments were separated by two days.

### 2.8. Von Frey Filament Test

Mice were habituated in in the plastic box (8 × 8 × 10 cm^3^) for 3 successive days. Before the test, mice were placed in arena ahead of the test until they calmed down. Then, a standard protocol was used to vertically stimulate the left hind paws of mice with a series of *von* Frey hairs in logarithmic increments of stiffness (0.07–2.0 g) as our previous reports [21]. Each *von* Frey hair was placed vertically on the sole of the hind paw for 4–5 s. The minimal force that caused responsive behaviors (lifting, flicking, or licking responses) 3 times out of 5 times of tests was regarded as the paw withdraw threshold of this animal (cut-off value is set to 2.0 g). 

### 2.9. Hargreaves Test

Mice were placed in a white plastic box on a glass floor. A radiant heat beam was focused on the hind paw. Each test was repeated 3 times with a minimum interval of 5 min. To avoid tissue damage, the cut-off latency for the test was 20 s. 

### 2.10. Hotplate Test

Mice were exposed to hot plate at temperatures of 48, 52 and 56 °C. The movement of the mice was restrained by a 20 cm diameter plexiglass cylinder. The latency of the withdrawal response such as licking, flicking, or lifting the hind paw was recorded, and the cut-off time was set to 30 s. Each temperature is tested only once, 10 min apart.

### 2.11. Open Field Test (OFT)

The OFT was used to evaluate the locomotor activity and the anxiety-related behavior of mice. Mice were placed in the central of a polystyrene enclosure (50 × 50 × 40 cm) and allowed to move freely for 15 min and were videotaped separately. The following mice were then swabbed with 75% ethanol before being tested. At the end of the test, an automated analysis system was used to quantify the total distance traveled, velocity and the time spent in the central area.

### 2.12. Elevated plus Maze (EPM) Test

The EPM is made of a white Plexiglas apparatus consisting of two opposing open arms (30 × 5 cm), two opposing closed arms (30 × 5 cm), and a central area (5 × 5 cm). The platform was 50 cm above the floor. In general, the mice were placed alone in the central area of the maze and allowed to explore for 9 min. The time spent in the open arms was recorded by the automated analysis system.

### 2.13. Formalin Test

After a 30 min habituation period, mice were subcutaneously injected with 4% formalin (20 µL) in the left paw, and mice behaviors were videotaped for 60 min. The paw licking and biting times were divided into 5 min sections. Additionally, the acute (0–10 min) and inflammatory (10–45 min) phase pain responses were quantified.

### 2.14. Neuropathic Pain Model

We used SNI to establish a neuropathic pain model [17]. Mice received general anesthesia with pentobarbital sodium (40 mg/kg, *i.p.*). After disinfection, three branches of the sciatic nerve are clearly exposed through small incisions in the skin and muscle. The common peroneal nerve and the tibial nerve were ligated with 5-0 thread without damaging the sural nerve. The nerve was then severed between ligation and the distal nerve stump was removed 1–2 mm. Finally, the myofascial membrane and skin were closed.

### 2.15. Statistical Analysis

All data are presented as mean ± s.e.m. We used Prism 8 software (GraphPad, San Diego, CA, USA) for statistics. The data were statistically analyzed with two-tailed *t-*test for the raw data, one-way or two-way ANOVA with repeated measures for multiple comparisons. *p* < 0.05 was considered statistically significant.

## 3. Results

### 3.1. The ZIR^GABA+^–PVT Pathway Involvement in Nociception

**Figure 1:**
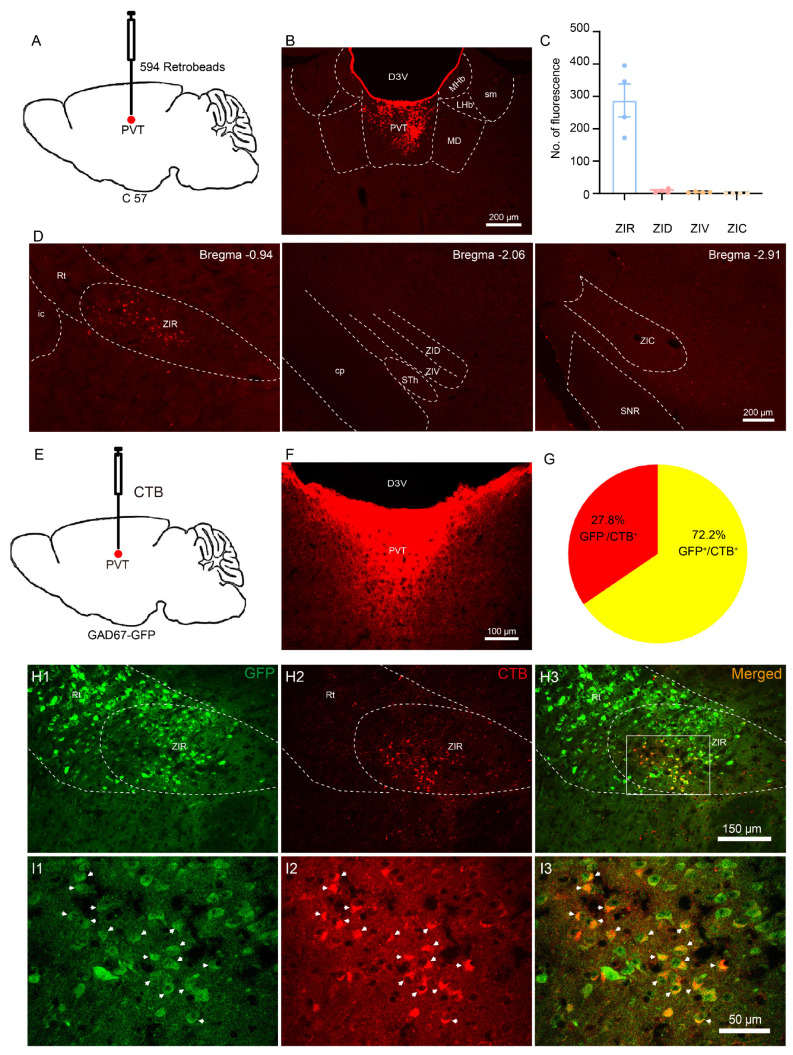
The type of neurons that ZI projects to PVT. (**A**) Schematic of strategy to inject retrograde tracer retroBeads into PVT. (**B**) The injection site of retroBeads in PVT. D3V: dorsal 3rd ventricle; MHb: medial habenular nucleus; LHb: lateral habenular nucleus; sm: stria medullaris; MD: mediodorsal thalamic nucleus. Scale bars = 200 μm. (**C**,**D**) The distribution of retroBead-labeled neurons in ZI. Rt: reticular nucleus; ic: internal capsule; cp: cerebral peduncle; STh: subthalamic nucleus; SNR: substantia nigra, reticular part. Scale bars = 200 μm. (**E**) Schematic of strategy to inject retrograde tracer CTB into PVT. (**F**) The injection site of CTB in PVT. Scale bars = 100 μm. (**G**) The ratios of CTB-labeled neurons were double-labeled with GFP in ZI of GAD67-GFP mice. (**H1**–**I3**) The distribution of GAD67-GFP neurons (green), the CTB-labeled neurons (red) and the white arrow heads indicate the GFP/CTB double-labeled neurons (yellow) in ZI. Scale bars = 150 μm. Magnified scale bar = 50 μm.

First, to determine the connections between ZI and PVT, we injected the retrograde tracer 594 retroBeads into PVT of C57BL/6J mice (Figure 1A). We found that the retrograde labeled neurons mainly occupied the ZIR and were rarely observed in the other parts of ZI (Figure 1C,D), which is consistent with previous study [19].

Next, to clarify the types of neurons that ZIR projects to PVT, we applied the retrograde tracer cholera toxin B subunit (CTB) into PVT of glutamic acid decarboxylase-green fluorescence protein (GAD67-GFP) knock-in mice (Figure 1E). We found that there were many GFP-labeled positive neurons in ZI of GAD67-GFP mice, consistent with the data for glutamic acid decarboxylase 2 (GAD2) mRNA distribution shown in the Allen Brain Atlas (http://www.brain-map.org) (accessed on 1 January 2023). The retrograde neurons double-labeled with CTB/GFP were observed in ZIR (Figure 1H1–I3). The results showed that approximately 72.2% of the CTB-labeled were co-expressed with GFP in ZIR (Figure 1G), suggesting that most neurons in ZIR project to PVT are GABAergic.

**Figure 2:**
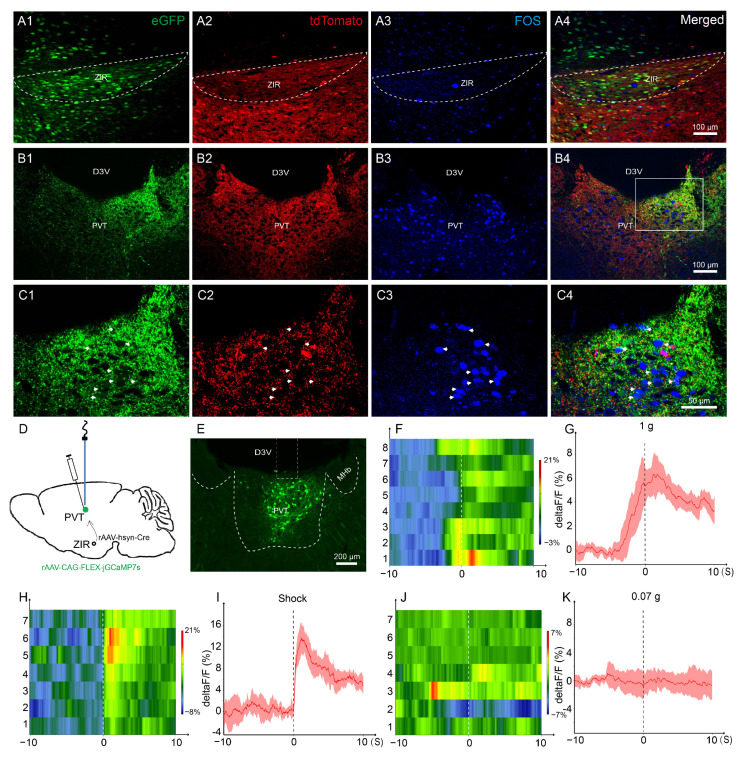
The ZIR^GABA+^–PVT pathway involvement in nociception. (**A1**–**A4**) The injection site of the rAAV-hSyn-eGFP (green), the expression of tdTomato-positive GAD2^+^ neurons (red) and the FOS-positive neurons (blue) in ZIR of the formalin-injected GAD2-Ai9 mice. Scale bars = 100 μm. (**B1**–**C4**) The distribution of anterogradely labeled axonal terminals (green) from ZIR, the expression of GABA terminals (red) and the FOS-positive neurons (blue) in PVT of the formalin-injected mice. The white arrowheads indicate eGFP/tdTomato/FOS triple-labeled neurons. Scale bars = 100 μm. Magnified scale bar = 50 μm. (**D**) Schematic showing the rAAV-hSyn-Cre injected into ZIR and the rAAV-CAG-FLEX-jGCaMP7s injected into PVT, and an optical fiber was placed above PVT. (**E**) The injection site of jGCaMP7s virus and the location of the optic fiber in PVT. Scale bar = 100 μm. (**F**,**G**) Heatmap and average Ca^2+^ transients of ZIR-projecting PVT neurons in mice receiving 1.0 g *von* Frey filament stimulation. (**H**,**I**) Heatmap and average Ca^2+^ transients of ZIR-projecting PVT neurons in mice receiving shock (0.3 mA, 0.5 s) stimulation. (**J**,**K**) Heatmap and average Ca^2+^ transients of ZIR-projecting PVT neurons in mice receiving 0.07 g *von* Frey filament stimulation.

We bred GAD2-cre mice with Ai9 reporter mice, in which the GAD2^+^ neurons were labeled with tdTomato. To investigate whether the ZIR–PVT pathway is involved in nociceptive transmission, we injected rAAV-hSyn-eGFP into the right ZIR of GAD2-Ai9 mice (Figure 2A1–A4). Three weeks later, the mice were applied with 4% formalin (20 μL) in the left hind paw [14], and perfused after 2 h. Fos protein (FOS) is widely used as a biomarker of early neuronal activation in relation to the pathophysiology of pain [22,23]. Immunohistochemical staining showed that in response to nociceptive stimuli, many FOS-positive neurons were observed in PVT (Figure 2B3). The anterogradely labeled axonal terminals (green) from the ZIR showed GAD2-positive (red), and made close connections with the FOS positive neurons (blue) in PVT (Figure 2C1–C4). These data indicate that the activated PVT neurons receives GABAergic projections from the ZIR in nociception.

**Figure 3:**
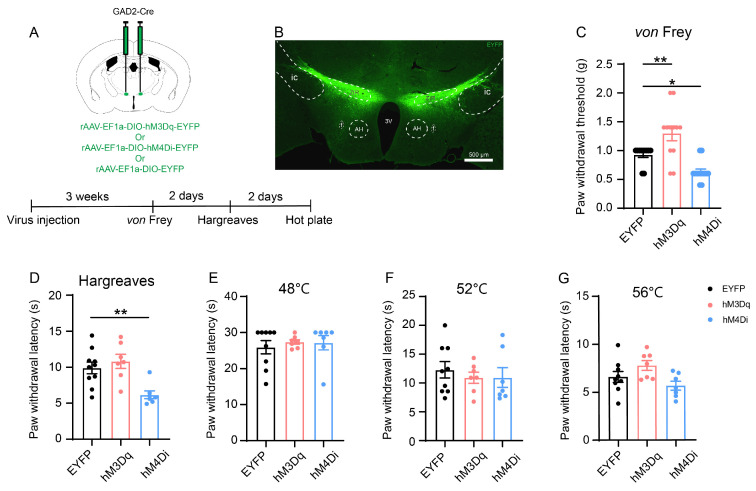
The roles of ZIR GABAergic neurons in nociception. (**A**) Schematic of bilateral ZIR injection of hM3Dq/hM4Di/EYFP virus to specifically manipulate the GABAergic neurons in GAD2-Cre mice and the timeline of behavioral experiment. (**B**) The injection site of the virus in ZIR. AH: anterior hypothalamic area; f: fornix. Scale bars = 500 μm. (**C**) Effects of chemogenetic manipulation of ZIR GABAergic neurons on mechanical hypersensitivity (F_2,33_ = 16.21, *p* < 0.0001), *n* = 11 for the EYFP group, *n* = 12 for the hM3Dq group, *n* = 13 for the hM4Di group. (**D**) Effects of chemogenetic manipulation of ZIR GABAergic neurons on heat hypersensitivity in Hargreaves test (F_2,21_ = 8.141, *p* = 0.0024), *n* = 10 for the EYFP group, *n* = 7 for the hM3Dq group, *n* = 7 for the hM4Di group. (**E**–**G**) Effects of chemogenetic manipulation of ZIR GABAergic neurons on heat thresholds in hotplate test, *n* = 9 for EYFP group, *n* = 7 for the hM3Dq group, *n* = 7 for the hM4Di group. ^*^ *p* < 0.05, ^**^ *p* < 0.01. One-way ANOVA with Dunnett’s multiple comparisons test for (**C**–**G**).

To further confirm the distinct relationships between the ZIR–PVT pathway and the pain-related responses, we applied in vivo fiber photometry recording to monitor their Ca^2+^ levels in free moving mice. We stereotaxically injected anterograde transmonovirus rAAV-hSyn-Cre into ZIR combined with the application of ultrasensitive protein calcium sensors (cre-dependent jGCaMP7s) into PVT, and implanted an optical fiber above PVT for long-term recordings of jGCaMP7s fluorescence for in vivo calcium imaging via fiber photometry (Figure 3D). We found an increase in the activities of ZIR neurons projecting to PVT following 1.0 g *von* Frey filament stimuli (Figure 2F,G) and shock (Figure 2H,I), whereas no significance in Ca^2+^ signals were detected by the non-nociception stimuli (0.07 g *von* Frey filament, Figure 2J,K), indicating that the activities of PVT neurons receiving afferents from ZIR were specifically evoked by nociceptive stimuli.

Taken together, our results indicate that in response to nociceptive stimuli, these activated PVT neurons receives GABAergic afferents from ZIR. Importantly, an in vivo fiber photometry recording indicated that the excitability of the ZIR^GABA+^–PVT pathway was increased in nociception.

### 3.2. In Situ Chemogenetic Activation of the ZIR GABAergic Neurons Attenuates but Inhibition of ZIR Promotes Nociceptive Behaviors

We observed that the ZIR^GABA+^–PVT pathway activated in nociception. Next, we characterized the functional roles of ZIR in nociceptive behaviors with the chemogenetic methods of designer receptors exclusively activated by designer drugs (DREADDs). The rAAV encoding the cre-dependent excitatory human M3 muscarinic receptor (hM3Dq) or inhibitory (hM4Di) designer receptor fused with enhanced yellow fluorescence protein (EYFP) were injected into bilateral ZIR of GAD2-Cre mice (Figure 3A). The control mice were injected with the rAAV-EF1a-DIO-EYFP (EYFP group). Three weeks later, at least 30 min after intraperitoneal injection of clozapine (0.1 mg/kg), behavioral data showed that compared with the EYFP group, the mechanical pain threshold was distinctly increased in the hM3Dq group (hM3Dq-Clozapine vs. EYFP-Clozapine: *p* = 0.0087). On the other hand, the mechanical pain threshold was decreased when chemogenetic inhibition of ZIR GABAergic neurons (hM4Di-Clozapine vs. EYFP-Clozapine: *p* = 0.0352, Figure 3C).

We also examined the thermal hyperalgesia in chemogenetic activation/inhibition. In Hargreaves test, the latency of the withdrawal thresholds in the hindpaw was significantly reduced in the hM4Di group after clozapine injection compared with the control group (hM4Di-Clozapine vs. EYFP-Clozapine: *p* = 0.0070, Figure 3D), whereas no significant difference was observed in the hM3Dq group (hM3Dq-Clozapine vs. EYFP-Clozapine: *p* = 0.6524). Another type of thermal nociception measured by hotplate test was not changed, indicating that thermal pain could be partial influenced by chemogenetic inhibition of the ZIR GABAergic neurons (48 °C: hM3Dq-Clozapine vs. EYFP-Clozapine: *p* = 0.7617, hM4Di-Clozapine vs. EYFP-Clozapine: *p* = 0.8220; 52 °C: hM3Dq-Clozapine vs. EYFP-Clozapine: *p* = 0.7247, EYFP-Clozapine vs. hM4Di-Clozapine: *p* = 0.7336; 56°C: hM3Dq-Clozapine vs. EYFP-Clozapine: *p* = 0.2236, hM4Di-Clozapine vs. EYFP-Clozapine: *p* = 0.3708, Figure 3E–G). 

Collectively, these data suggest that chemogenetic activation of ZIR GABAergic neurons is sufficient to induce antinociceptive effect, whereas inhibition of ZIR GABAergic neurons results in mechanical and partial heat hypersensitivity.

### 3.3. Optogenetic Activation of the ZIR^GABA+^–PVT Pathway Alleviates Nociception but Inhibition Induces Hyperalgesia

**Figure 4:**
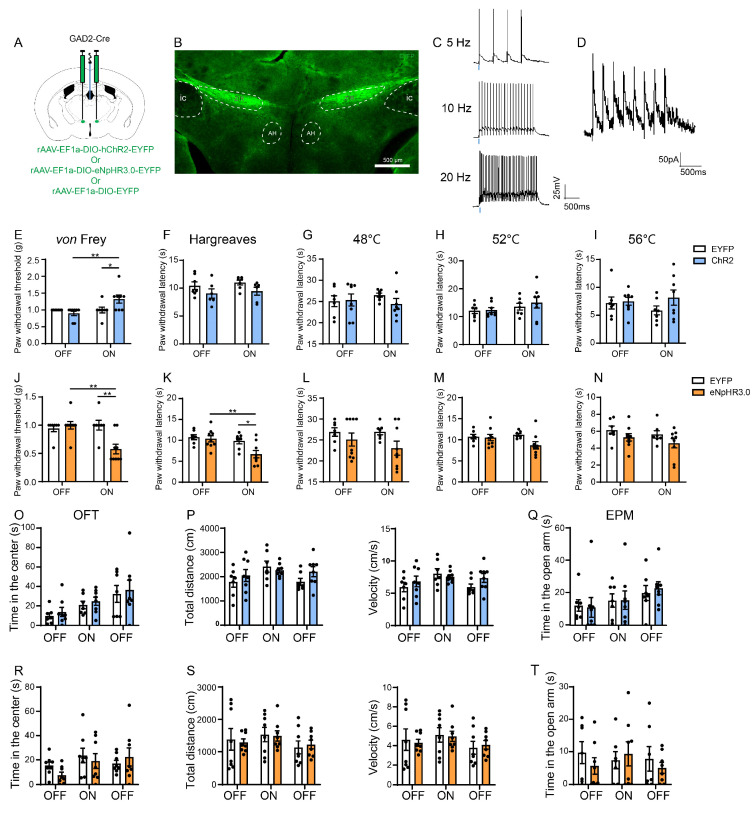
Optogenetic manipulation of the ZIR^GABA+^–PVT pathway in nociception. (**A**) Schematic of bilateral ZIR injection of hChR2/eNpHR3.0/EYFP virus and the optical fiber was placed above PVT to specifically manipulate the ZIR^GABA+^–PVT pathway. (**B**) The injection site of the virus in ZIR. Scale bars = 500 μm. (**C**) The 473 nm laser-induced time-locked action potential firing at 5 Hz, 10 Hz and 20 Hz in ChR2-expressing neurons in ZIR. Scale bars = 500 ms, 25 mV. (**D**) The blue laser pulse-evoked IPSC in PVT. Scale bars = 500 ms, 50 pA. (**E**) Effects of ZIR^GABA+^–PVT pathway photoactivation on mechanical thresholds (Interaction F_1,26_ = 6.470, *p* = 0.0173), *n* = 7 for the EYFP group, *n* = 8 for the ChR2 group. (**F**) Effects of ZIR^GABA+^–PVT pathway photoactivation on heat thresholds in Hargreaves test (Interaction F_1,22_ = 0.01312, *p* = 0.9098), *n* = 7 for the EYFP group, *n* = 6 for the ChR2 group. (**G**–**I**) Effects of the ZIR^GABA+^–PVT pathway photoactivation on heat thresholds in hotplate test (48 °C: Interaction F_1,26_ = 0.9245, *p* = 0.3451; 52 °C: Interaction F_1,26_ = 0.2022, *p* = 0.6567; 56 °C: Interaction F_1,26_ = 0.9263, *p* = 0.3447), *n* = 7 for the EYFP group, *n* = 8 for the ChR2 group. (**J**) Effects of ZIR^GABA+^–PVT pathway photosilencing on mechanical hypersensitivity (Interaction F_1,28_ = 9.799, *p* = 0.0041), *n* = 7 for the EYFP group, *n* = 9 for the eNpHR3.0 group. (**K**) Effects of the ZIR^GABA+^–PVT pathway photosilencing on heat hypersensitivity in Hargreaves test (Interaction F_1,28_ = 3.208, *p* = 0.0841), *n* = 7 for the EYFP group, *n* = 9 for the eNpHR3.0 group. (**L**–**N**) Effects of the ZIR^GABA+^–PVT pathway photosilencing on heat thresholds in hotplate test (48 °C: Interaction F_1,28_ = 0.5525, *p* = 0.4635; 52 °C: Interaction F_1,28_ = 0.2511, *p* = 0.1243; 56°C: Interaction F_1,28_ = 0.03799, *p* = 0.8469), *n* = 7 for the EYFP group, *n* = 9 for the eNpHR3.0 group. (O–Q) Effects of ZIR^GABA+^–PVT pathway photoactivation on locomotor abilities and anxiety-like behaviors in open field test and the elevated plus-maze test, *n* = 7 for the EYFP group, *n* = 8 for the ChR2 group. (R-T) Effects of the ZIR^GABA+^–PVT pathway photosilencing on locomotor abilities and anxiety-like behavior in open field test and the elevated plus-maze test. OF: *n* = 8 for the EYFP group, *n* = 8 for the eNpHR3.0 group; EPM: *n* = 7 for the EYFP group, *n* = 8 for the eNpHR3.0 group. ^*^ *p* < 0.05, ^**^ *p* < 0.01. Two-way ANOVA with Tukey’s multiple comparisons test for (**A**–**T**).

Our previous results suggested an essential role of in situ ZIR GABAergic neurons in nociception, next we aimed to observe the effects of the pathway from the ZIR GABAergic neurons to PVT on nociceptive neurotransmission by optogenetic manipulations. We injected rAAV-EF1a-DIO-ChR2-EYFP and rAAV-EF1a-DIO-eNpHR3.0-EYFP into ZIR of GAD2-Cre mice (Figure 4A). The rAAV-EF1α-DIO-EYFP were injected as the control. Three weeks later, the opto fibers were implanted above PVT. 

Above all, the effectiveness of the ChR2 virus by the whole-cell current-clamp recordings was examined. The brain slices were performed using 5 Hz, 10 Hz, and 20 Hz pulse trains stimulation, results showed that the blue laser pulses induced time-locked action potential firing in the neurons expressing ChR2-EYFP in ZIR (Figure 4C). Moreover, the blue laser pulse significantly induced inhibitory postsynaptic currents (IPSC) in PVT (Figure 4D). These results proved that the PVT neurons could be strongly inhibited by ZIR stimulation. 

Behavioral data showed that optogenetic activation of the ZIR^GABA+^–PVT pathway with blue laser light (473 nm, 20 Hz, 5–10 mW) induced a significantly increase in the threshold of mechanical pain (Light on vs. Light off in the ChR2 group: *p* = 0.0050; EYFP vs. ChR2 in the Light-on state: *p* = 0.0492). The mice received the virus without ChR2 expression did not show any difference when the light was on (Light on vs. Light off in the EYFP group: *p* > 0.9999, Figure 4E). There was no significant effect in Hargreaves test (Light on vs. Light off in the ChR2 group: *p* = 0.9989; EYFP vs. ChR2 in the Light-on state: *p* = 0.5166, Figure 4F) or hotplate test when activation of ZIR^GABA+^–PVT pathway (48 °C: Light on vs. Light off in the ChR2 group: *p* = 0.9460, EYFP vs. ChR2 in the Light-on state: *p* = 0.6279; 52 °C: Light on vs. Light off in the ChR2 group: *p* = 0.4942, EYFP vs. ChR2 in the Light-on state: *p* = 0.8647; 56 °C: Light on vs. Light off in the ChR2 group: *p* = 0.9660, EYFP vs. ChR2 in the Light-on state: *p* = 0.4112, Figure 4G–I). 

Inversely, optogenetic inhibition of the ZIR^GABA+^–PVT pathway with yellow light (589 nm, 5 mW) induced mechanical hypersensitivity (Light on vs. Light off in the eNpHR3.0 group: *p* = 0.0016; EYFP vs. eNpHR3.0 in the Light-on state: *p* = 0.0033, Figure 4J). Inhibition of the ZIR^GABA+^–PVT pathway also significantly decreased the latency withdrawal thresholds in Hargreaves test (Light on vs. Light off in the eNpHR3.0 group: *p* = 0.0063; EYFP vs. eNpHR3.0 in the Light-on state: *p* = 0.0356, Figure 4K). However, the results from hotplate test did not show any significant differences (48 °C: Light on vs. Light off in the eNpHR3.0 group: *p* = 0.8614, EYFP vs. eNpHR3.0 in the Light-on state: *p* = 0.8614; 52 °C: Light on vs. Light off in the eNpHR3.0 group: *p* = 0.2356, EYFP vs. eNpHR3.0 in the Light-on state: *p* = 0.0901; 56 °C: Light on vs. Light off in the eNpHR3.0 group: *p* = 0.6646, EYFP vs. eNpHR3.0 in the Light-on state: *p* = 0.4353, Figure 4L–N). 

The general locomotor abilities in the open field test (OFT, total distance and velocity: EYFP vs. ChR2 in the Light-on state: *p* = 0.9940, EYFP vs. eNpHR3.0 in the Light-on state: *p* > 0.9999) and the anxiety-like behaviors evaluate by the elevated plus maze test (EPM) test were not affected during the optogenetic manipulations of the ZIR^GABA+^–PVT pathway (center time in OFT: EYFP vs. ChR2 in the Light-on state: *p* = 0.9982, EYFP vs. eNpHR3.0 in the Light-on state: *p* = 0.9859; time in open arm: EYFP vs. ChR2 in the Light-on state: *p* > 0.9999, EYFP vs. eNpHR3.0 in the Light-on state: *p* = 0.9974, Figure 4O–T). 

Therefore, our findings demonstrate that the specific activation of the ZIR^GABA+^–PVT pathway alleviates mechanical hyperalgesia. However, inhibition of the ZIR^GABA+^–PVT pathway is sufficient to evoke mechanical hyperalgesia and but partial heat hyperalgesia.

### 3.4. Chemogenetic Activation of the ZIR^GABA+^–PVT Pathway Attenuates Inflammatory Pain and Neuropathic Pain

**Figure 5:**
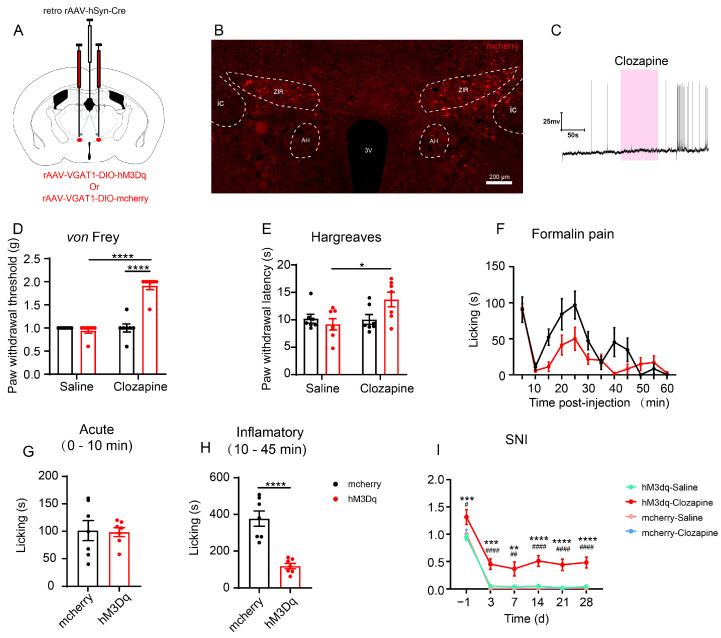
Chemogenetic activation of the ZIR^GABA+^–PVT pathway relieves inflammatory and neuropathic pain. (**A**) Schematic of bilateral ZIR injection of rAAV-DIO-VGAT1-hM3Dq-mcherry and rAAV- VGAT1-DIO-mcherry, and the retrograde virus were injected into PVT simultaneously to specifically activate the ZIR^GABA+^–PVT pathway. (**B**) The injection site of the virus in ZIR. Scale bars = 200 μm. (**C**) Whole-cell current-clamp recordings on hM3Dq-expressing ZIR–PVT projection neurons. Bath application of 20 μM clozapine caused an action potential firing. Scale bars = 50 s, 25 mV. (**D**) Chemogenetic activation of the ZIR^GABA+^–PVT pathway on mechanical thresholds (Interaction F_1,24_ = 51.76, *p* < 0.0001), *n* = 7. (**E**) Chemogenetic activation of the ZIR^GABA+^–PVT pathway on heat thresholds in Hargreaves test (Interaction F_1,24_ = 5.076, *p* = 0.0337), *n* = 7. (**F**) The time spent licking paws within 60 min after formalin injection. (**G**) Time spent licking paws during the acute phase of formalin test (t_12_=0.1456), *n* = 7. (**H**) Time spent licking paws during the inflammatory phase of formalin test (t_12_ = 5.864), *n* = 7. (**I**) Chemogenetic activation of the ZIR^GABA+^–PVT pathway significantly increased withdrawal threshold in SNI mice (Interaction F_15,120_ = 0.5456, *p* = 0.9095), *n* = 7. ^*^ *p* < 0.05, ^**^ *p* < 0.01, ^***^ *p* < 0.001, ^****^ *p* < 0.0001, compared with the hM3Dq-Saline group. ^#^ *p* < 0.05, ^##^ *p* < 0.01, ^####^ *p* < 0.0001, compared with the mcherry-Clozapine group. Unpaired *t-*test for (**G**,**H**). Two-way ANOVA with Tukey’s multiple comparisons test for (**D**,**E**). Two-way ANOVA with Sidak’s multiple comparisons test for (**I**).

Our previous results indicated that the ZIR^GABA+^–PVT pathway plays a role in nociceptive neurotransmission. Subsequently, it is important to verify the effects of the ZIR^GABA+^–PVT pathway in pain models. After stereotaxically injected the cre-dependent and GABAergic neuronal promoter vesicular GABA transporter 1 (VGAT1) virus (rAAV-VGAT1-DIO-hM3Dq-mcherry) into ZIR of C57BL/6J mice (rAAV-VGAT1-DIO-mcherry as the control) along with the retrograde virus injection into PVT (Figure 5A). The effectiveness of the virus was examined by the whole-cell current-clamp recordings from brain slices, results showed that a bath application of 20 μM clozapine-induced robust action potential firing in ZIR (Figure 5C).

Intraperitoneal injection of clozapine specifically activated the ZIR^GABA+^–PVT pathway, the mechanical hypersensitivity of mice decreased significantly (hM3Dq-Saline vs. hM3Dq-Clozapine: *p* < 0.0001; hM3Dq-Clozapine vs. mcherry-Clozapine: *p* < 0.0001) and heat in Hargreaves test (hM3Dq-Saline vs. hM3Dq-Clozapine: *p* = 0.0240; hM3Dq-Clozapine vs. mcherry-Clozapine: *p* = 0.0868, Figure 5D,E). 

In formalin-induced inflammatory pain model, compared with the control group, activation of the ZIR^GABA+^–PVT pathway significantly shortened the time spent on licking paws in the inflammatory phase (second phase, 10–45 min) but not the acute phase (first phase, 0–5 min, acute: hM3Dq-Clozapine vs. mcherry-Clozapine: *p* = 0.8867; inflammatory: hM3Dq-Clozapine vs. mcherry-Clozapine: *p* < 0.0001, Figure 5F–H). The duration of paw licking decreased from 377.3 ± 41.7 s to 118.1 ± 14.7 s. The results demonstrate that the activation of the ZIR^GABA+^–PVT pathway might alleviate formalin-induced inflammatory pain in mice.

Moreover, as a classical neuropathic pain model, spared nerve injury model (SNI) was performed in our study. There was a significant increase in mechanical pain threshold when activation of the ZIR^GABA+^–PVT pathway in the SNI group (Day-1: hM3Dq-Saline vs. hM3Dq-Clozapine: *p* = 0.0009, hM3Dq-Clozapine vs. mcherry-Clozapine: *p* = 0.0117; Day3: hM3Dq-Saline vs. hM3Dq-Clozapine: *p* = 0.0003, hM3Dq-Clozapine vs. mcherry-Clozapine: *p* < 0.0001; Day7: hM3Dq-Saline vs. hM3Dq-Clozapine: *p* = 0.0077, hM3Dq-Clozapine vs. mcherry-Clozapine: *p* = 0.0024; Day14: hM3Dq-Saline vs. hM3Dq-Clozapine: *p* < 0.0001, hM3Dq-Clozapine vs. mcherry-Clozapine: *p* < 0.0001; Day21: hM3Dq-Saline vs. hM3Dq-Clozapine: *p* < 0.0001, hM3Dq-Clozapine vs. mcherry-Clozapine: *p* < 0.0001; Day28: hM3Dq-Saline vs. hM3Dq-Clozapine: *p* < 0.0001, hM3Dq-Clozapine vs. mcherry-Clozapine: *p* < 0.0001, Figure 5I). These findings suggest that the activation of the ZIR^GABA+^–PVT pathway could decrease mechanical pain in neuropathic pain.

Taken together, chemogenetic activation of the ZIR^GABA+^–PVT pathway might exert analgesic effects in inflammatory pain and neuropathic pain in mice.

### 3.5. The ZIR^GABA+^–PVT Pathway Modulates Nociception through GABA-A Receptor (GABA_A_R)-Expressing Neurons in PVT

**Figure 6:**
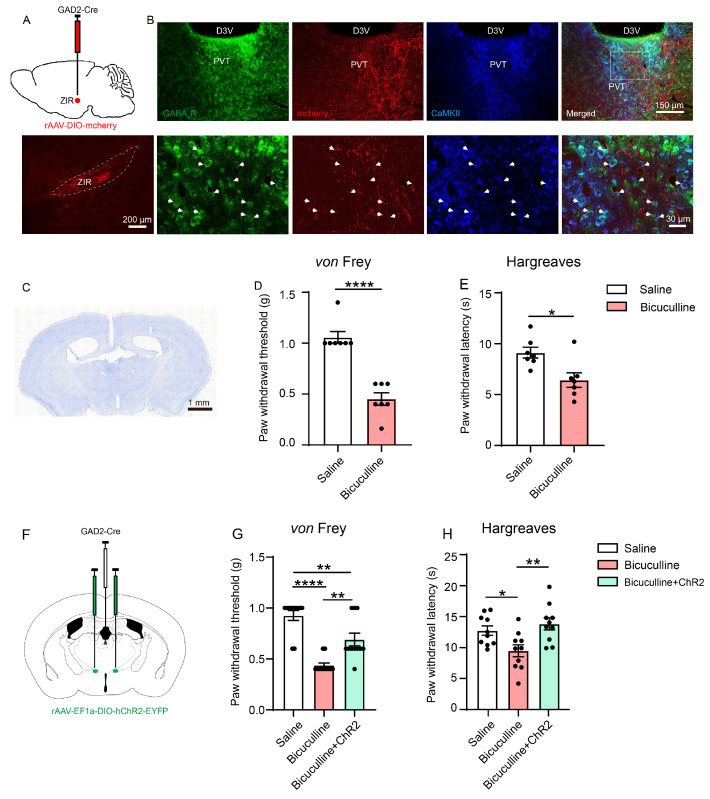
Optogenetic and pharmacological manipulation of the ZIR^GABA+^–PVT pathway. (**A**) Schematic of virus injection and the injection site of the rAAV-DIO-mcherry in the right ZIR of GAD2-Cre mice. Scale bars = 200 μm. (**B**) Images of GABA_A_R, mcherry and CaMKII expressions in PVT. The white square displayed as a triple-staining of GABA_A_R (green), mcherry (red) and CaMKII (blue). Scale bar = 150 μm (**B**, top), and 30 μm (**B**, bottom). (**C**) Nissl’s staining of the cannula implantation regions. Scale bars = 1 mm. (**D**) Effects of microinjection of GABA_A_R antagonists bicuculline on mechanical thresholds (t_12_ = 7.212), *n* = 7. (**E**) Effects of microinjection of GABA_A_R antagonists bicuculline on heat thresholds in Hargreaves test (t_12_ = 3.031), *n* = 7. (**F**) Schematic diagram of the experimental approach combining optogenetics with local pharmacology. (**G**) Effects of optogenetic and pharmacological manipulation of the ZIR^GABA+^–PVT pathway on mechanical thresholds (F_2,30_ = 26.30, *p* < 0.0001), *n* = 11. (**H**) Effects of optogenetic and pharmacological manipulation of the ZIR^GABA+^–PVT pathway on heat thresholds in Hargreaves test (F_2,27_ = 6.284, *p* = 0.0057), *n* = 10. ^*^ *p* < 0.05, ^**^ *p* < 0.01, ^****^ *p* < 0.0001. Unpaired *t-*test for (**D**,**E**). One-way ANOVA with Tukey’s multiple comparisons test for (**G**,**H**).

Since the neurons from ZIR project to PVT were mainly GABAergic neurons, the underlying mechanism of the ZIR^GABA+^–PVT pathway in nociceptive modulation is needed to be specified in detail. Therefore, we injected cre-dependent anterograde virus (rAAV-DIO-mcherry) into the right ZIR of GAD2-Cre mice (Figure 6A). Calcium-calmodulin dependent protein kinase II (CaMKII) is generally considered to be a marker for excitatory neurons in thalamus [24]. Herein, we performed theCaMKII/GABA_A_R double-labeled immunostaining in GAD2-Cre mice receiving rAAV-DIO-mcherry injection into ZIR. Results showed that the CaMKII/GABA_A_R double-labeled neurons densely distributed in PVT (Figure 6B), indicating these excitatory neurons expressing GABA_A_R. Furthermore, the anterogradely labeled axonal terminals (red) from the ZIR made close connections with GABA_A_R/CaMKII double-labeled neurons in PVT (Figure 6B). The immunostainings indicate that the inhibitory effects of GABAergic terminals from ZIR^GABA+^ to glutamatergic PVT neurons might be mediated by GABA_A_R located at PVT.

We next detected the effects of GABA_A_R on PVT in nociceptive neurotransmission by locally microinjected GABA_A_R antagonists bicuculline (0.1 μg) in PVT with saline infused as control. The cannula implantation regions were confirmed at the end of all behavioral tests (Figure 6C). We found that pharmacological inhibition of GABA_A_R in PVT decreased the mechanical pain threshold and thermal hyperplasia (*von* Frey test: Bicuculline vs. Saline: *p* < 0.0001; Hargreaves test: Bicuculline vs. Saline: *p* = 0.0104, Figure 6D,E), revealing that the GABA inhibition of PVT via GABA_A_R potentially contributes to pain hypersensitivity.

To further confirm the roles of GABA_A_R in the ZIR^GABA+^–PVT pathway, we injected rAAV-EF1a-DIO-ChR2-EYFP into ZIR (Figure 6F). After microinjection of bicuculline for 20 min, blue laser stimulation was performed. Results demonstrated that bicuculline decreased the mechanical pain threshold, which could be mostly rescued by activating the ZIR^GABA+^–PVT pathway (Bicuculline vs. Saline: *p* < 0.0001; Bicuculline + ChR2 vs. Bicuculline: *p* = 0.0021; Bicuculline + ChR2 vs. Saline: *p* = 0.0042, Figure 6G). This effect was also found in the Hargreaves test (Bicuculline vs. Saline: *p* = 0.0423; Bicuculline + ChR2 vs. Bicuculline: *p* = 0.0057, Figure 6H). The significant increase in pain threshold from 0.40 ± 0.02 g (Bicuculline group) to 0.69 ± 0.06 g (Bicuculline + ChR2 group) in the *von* Frey test and 9.48 ± 0.96 s (Bicuculline group) to 13.83 ± 0.97 s (Bicuculline + ChR2 group) in the Hargreaves test. These findings demonstrate that GABA_A_R is crucial for GABAergic inhibition from ZIR to PVT pathway in nociceptive neurotransmission.

Therefore, our results suggest that the ZIR^GABA+^–PVT pathway plays a critical role in regulating nociceptive neurotransmission through GABA_A_R-expressing glutamatergic neurons in PVT.

## 4. Discussion

In this study, we present that the ZIR^GABA+^–PVT pathway is involved in nociceptive neurotransmission. Our study found that these PVT neurons which received ZIR projections were activated by nociceptive stimulation. Furthermore, we confirmed that activation of ZIR GABAergic neurons contribute to antinociception, whereas inhibition of ZIR induce mechanical hypersensitivity and partial heat hyperalgesia. Moreover, we also found that the activated the ZIR^GABA+^–PVT pathway alleviated nociception, whereas when inhibited, this pathway induced hyperalgesia, and these effects were acted through GABA_A_R-expressing neurons in PVT. 

GABA is an important inhibitory neurotransmitter in the central nervous system (CNS) and is essential for the balance between neuronal excitation and inhibition [25,26]. Excitation and inhibition imbalance in the activity of the thalamus is thought to be one of the important causes of pain [27]. Importantly, ZI has unidirectional feedforward inhibition control on other nuclei which are enriched excitatory neurons in thalamus, leading to the rapid and strong effects on neurotransmission of thalamus [28,29]. For instance, the pathological increased activity of mediodorsal thalamus (MD) and the posterior complex of the thalamus (PO) activity after spinal cord injury are associated with ZI inhibition [30,31]. This evidence suggests that ZI plays a key role in the inhibitory modulation of the excitation and inhibition balance in thalamus. However, there are still few studies on the involvement of ZI inhibition in thalamus in nociceptive neurotransmission. 

As an important component of the midline thalamic nuclei, the PVT consists primarily of excitatory glutamatergic neurons that are thought to relay pain information across various cortical and subcortical regions [32]. Previous studies showed an increased c-Fos protein expression and phosphorylation of extracellular signal-regulating kinases (pERK) within PVT in hyperalgesic mice [33]. The hyperexcitation of electrophysiological characteristics of PVT neurons was observed in spinal nerve ligation (SNL) mice [34], indicating that the excitation and inhibition balance of PVT neurons plays an important role in pain, and inhibition of abnormal excitation of PVT might be essential for the occurrence and development of pain. In the present study, our results suggest that the ZIR^GABA+^–PVT pathway participates in nociceptive neurotransmission and the enhanced inhibitory inputs from ZIR to PVT reduced pain.

GABAergic signaling pathway is crucial for the neural circuit in nociceptive neurotransmission by acting on GABA_A_R [35,36]. For instance, microinjection of the GABA_A_R agonist muscimol into the ventrobasal thalamus (VB) relieves thermal hyperalgesia in chronic inflammatory pain [37], and antagonists of the GABA_A_R bicuculline applied to the dorsal medial prefrontal cortex (dmPFC) could produce analgesic effects [38]. In addition, a recent study revealed that the injection of muscimol into PVT effectively relieve mechanical pain in SNL mice [34]. We provide morphological and behavioral evidence that the distribution of GABA_A_R at the ZIR^GABA+^–PVT pathway is crucial in nociceptive neurotransmission. These findings show that activation of GABA_A_R in PVT might be a useful therapeutic strategy for antinociception. 

The connections between different sectors or neuron types contribute to multiple roles of ZI in physiological functions [39,40,41]. For example, The ZIV GABAergic neurons have the opposite effects to ZIV parvalbumin (PV)-positive neurons on pain modulation [14]. The activated neural circuit from the central amygdala neurons expressing protein kinase C-delta (PKCδ) to ZIV-GABAergic cells produces pain hypersensitivity in mice [42]. The A13 dopamine neurons in the middle of ZI are rapidly activated by acute nociceptive stimuli [43]. Our behavioral data provide evidence that ZIR is another important region that inhibition of GABAergic ZIR neurons induced behavioral hyperalgesia whereas activation of GABAergic ZIR neurons induced antinociception. However, in our study, we also admit that the virus injections might have additional impacts on other ZI regions except for ZIR, but the center of virus injection was in ZIR. Intriguingly, the somatostatin (SOM), calretinin (CR), and vesicular glutamate transporter-2 (VgluT2) expressing cells in ZIR encoded and modulated different components of anxiety [44]. It suggests that the different subpopulations of ZIR project to PVT may play more complex roles in pain, such as pain-related affective aspects, which are worth for our further exploration.

The effects of the ZIR^GABA+^–PVT pathway on different forms of pain stimulation increase the complexity of the neural circuit in processing pain sensation. As shown in the present study, the modulations by chemogenetic and optogenetic methods in ZIR^GABA+^ or the ZIR^GABA+^–PVT pathway mainly influence on mechanical hyperalgesia and thermal nociception induced by Hargreaves test. However, in hotplate test, another behavioral test for evaluating thermal nociception, results showed that there was no significance between groups by manipulations of ZIR^GABA+^ or ZIR^GABA+^–PVT. This may be associated with fact that the mice were free to move when the hotplate test was conducted, thus influenced by other components, such as defensive and freezing behaviors. Previous research has shown that ZIR is engaged in modulating defense behaviors and freezing behaviors [45]. These findings indicate ZIR is an integrative node that modulates multiple behaviors. Taken together, our results reveal the function of the ZIR^GABA+^–PVT pathway in modulating nociceptive neurotransmission.

In summary, we employed morphological, fiber optic recording, behavioral and other methods to clarify the roles of ZIR^GABA+^–PVT pathway in the regulation of nociceptive neurotransmission. 

## 5. Conclusions

It is universally acknowledged that pain remains a severe global health problem. The nature of neural circuits underlying the diverse components of the complex, multidimensional experience of pain is not well understood. Our study aims to investigate the circuit connectivity from the inhibitory ZIR^GABA+^ to excitatory PVT in pain using multiple techniques and methods, suggesting that the endogenous GABAergic inhibition is essential for excitation and inhibition balance in neurotransmission, which might be involved in nociception. Nowadays, the knowledge of neural connectivity and their dynamic regulation via functional structural plasticity has been confirmed that provide the beneficial effects for forward translation back to human therapies, for instance, neurostimulation and neuromodulation [1]. Based on the advanced knowledge for neural circuits, deep brain stimulation implantation surgery is an established treatment modality for certain CNS disorders, such as chronic pain [46]. Therefore, our findings can potentially provide a strategy for using of clinical approaches targeting specific neural circuits for pain management in future.

## Data Availability

The data presented in this study are available on request from the corresponding author. The data are not publicly available due to the privacy of other scientific research.
